# Challenges of Estimates in Drug-Related Overdose Deaths in Iran: Evidence from the Literature

**DOI:** 10.18502/ijph.v49i8.3914

**Published:** 2020-08

**Authors:** Mehran ROSTAMI, Shahab REZAEIAN

**Affiliations:** 1. Deputy of Health, Kermanshah University of Medical Sciences, Kermanshah, Iran; 2. Infectious Diseases Research Center, Kermanshah University of Medical Sciences, Kermanshah, Iran; 3. Clinical Research Development Center, Imam Reza Hospital, Kermanshah University of Medical Sciences, Kermanshah, Iran

## Dear Editor-in-Chief

Iranian Death Registration System (DRS) has become an ongoing process in recent years, but despite this progress, the Iranian DRS had several shortcomings (1, 2). Several factors affecting morbidity and mortality data collected by the Iranian DRS. For example, in Tehran, the capital of Iran, the mortality data were registered by Behesht-e-Zahra cemetery from 1995 to 2010, as well as in Isfahan, a central province of Iran, the mortality data were registered by Bagh-e-Rezvan Cemetery from 2007 to 2010 (2). Moreover, Alborz Province was separated from Tehran Province in 2011 which many cases of death related to this province were registered in Tehran Province earlier than 2011 (2).

Overall, the following issues have affected the Iranian DRS: inconsistency in DRS administration, duplicate recording of deaths, misclassification, geographical misalignment, incompleteness, and missing values (1, 2). For example, in a study performed using drug-related overdose deaths reported by the Iranian Ministry of Health and Medical Education (MoHME) in 2006 and 2011, the data were missing from the Alborz and Tehran provinces (3); this issue had adverse effect for conducting analysis and interpretation of data. Moreover, the environmental factors affecting central provinces such as Tehran and Alborz with rapid urbanization, high population density, receiving migrants from other parts of the country, and expansion of informal settlements may intensify illicit drug use epidemic (4). Furthermore, consumption patterns, route of drug consumption, and consumed drug-type had considerable effect on spread of illicit drug morbidity and mortality (5).

In Iran, the number of years lost due to disability (YLD)- for both sexes combined-caused by drug use disorders has dramatically increased between 1990 and 2010 (6). The rates of drug-related mortality are significantly higher among Iranian men compared to women (3, 4, 6, 7). Since women are perceived as the cornerstone of the family in Iranian culture, then substance use in women is considered a violation of this moral value (4, 5). In addition, illicit drug use among women is often under-reported or misclassified because of pressures resulted from socio-cultural context (3, 8). Moreover, barriers to accessing addiction care and treatment among women, due to the high stigma of drug use and limited number of women-only substance use services, increase the likelihood of illicit drug-related mortality (3, 4). According to forensic evidence, this claim is supported by forecasting that the overall temporal trend of drug-related overdose deaths are increasing among Iranian women (4).

Generally, the estimated drug-related overdose deaths in Iran are inaccurate given that several organizations collect data in various ways and sources of data collection are not consistent across the country. Socio-cultural limitations, different sources, and approaches of data collection complicate estimating overdose-related deaths as well as monitoring the trend of fatal drug overdoses over time.

Iran’s DRS could benefit from promoting collaborative efforts between all stakeholders involved with recording mortality data in Iran. Establishing a network including MoHME, forensic medicine organization, and civil registration systems would facilitate data linkage across different organizations. This could lead to smarter usage of the available resources, avoiding parallel activities, and providing a real estimation of the burden of drug-related overdose deaths across the country.
